# Prevalence of prediabetes and associated factors in southwest iran: results from Hoveyzeh cohort study

**DOI:** 10.1186/s12902-022-00990-z

**Published:** 2022-03-19

**Authors:** Seyed Jalal Hashemi, Majid Karandish, Bahman Cheraghian, Maryam Azhdari

**Affiliations:** 1 School of Medicine, Alimentary Tract Research Center, Clinical Sciences Research Institute, Ahvaz Jundishapur University of Medical Sciences, Ahvaz, Iran; 2 Nutrition and Metabolic Diseases Research Center, Clinical Sciences Research Institute, Ahvaz Jundishapur University of Medical Sciences, Ahvaz, Iran; 3 Department of Biostatistics and Epidemiology, School of Public Health, Alimentary Tract Research Center, Clinical Sciences Research Institute, Ahvaz Jundishapur University of Medical Sciences, Ahvaz, Iran; 4 Department of Nutrition, School of Public Health, Shahid Sadoughi University of Medical Sciences and Health Services, Yazd, Iran

**Keywords:** Prediabetes, Prevalence, Risk factors, Lipid profiles, Hypertension, Lifestyle, PERSIAN Cohort, Hoveyzeh

## Abstract

**Background:**

Increasing trend of prediabetes and diabetes is a global public health issue. On the other hand, prediabetes can increase the risk of developing some non-communicable diseases, including type 2 diabetes, cardiovascular disease, hypertension, fatty liver disease, etc. Given that there are modifiable various risk factors for prediabetes, this cross-sectional study aimed to evaluate the prevalence of prediabetes and its risk factors among adults.

**Methods:**

The present study included the baseline data from the Prospective Epidemiological Research Studies of the Iranian Adult and Neonates (PERSIAN), Hoveyzeh Cohort Study (*N* = 10,009). The demographic data, lifestyle habits, anthropometric data, and clinical and biochemical parameters were gathered. The odds ratio of prediabetes was assessed by logistic regression.

**Results:**

The final analysis was conducted on 7629 participants. The prevalence of overweight (36.7%), obesity (37.5%), prediabetes (30.29%), abnormal high density lipoprotein (35.4%), cholesterol (33.8%) and triglyceride (39.7%), and HTN (21.3%) were common. In the adjusted analysis, there were higher odds of having prediabetes for overweight (OR = 1.9, 95% CI: (1.19, 3.03), *p* = 0.007), obesity (OR = 3.18, 95% CI: (1.99, 5.07), *p* < 0.001), waist circumstance (WC) (OR = 1.024, 95% CI: (1.002, 1.03), *p* < 0.001), hip circumstance (HC) (OR = 1.01, 95% CI: (1.003, 1.02), *p* = 0.008), older age (OR = 1.04, 95% CI: (1.04, 1.05), *p* < 0.001), hypertension (OR = 1.38, 95% CI: (1.21, 1.57), *p* < 0.001),), glutamic-pyruvic transaminase (OR = 1.013, 95% CI: (1.007, 1.019), *p* = 0.001), glutamic-oxaloacetic transaminase (OR = 1.01, 95% CI: (1.006, 1.013), *p* < 0.001), triglyceride = 150–199 mg/dl (OR = 1.32, 95% CI: (1.16, 1.51), *p* < 0.001), triglyceride ≥ 200 mg/dl (OR = 1.64 (95% CI: 1.44, 1.86), *p* < 0.001), cholesterol = 200- 239 mg/dl (OR = 1.33, 95% CI: (1.18, 1.49), *p* < 0.001), and cholesterol ≥ 240 mg/dl (OR = 2.04, 95% CI: (1.72, 2.42), *p* < 0.001) in general population.

**Conclusion:**

The prevalence of prediabetes, overweight, obesity, HTN, and dyslipidemia was common. The greater chances of prediabetes were related to aging, overweight, obesity, HTN, higher liver enzymes, HC, abnormal WC, and dyslipidemia. It seems that practical interventions are necessary to prevent prediabetes.

**Supplementary Information:**

The online version contains supplementary material available at 10.1186/s12902-022-00990-z.

## Background

Diabetes is one of the major public health concerns and challenges for health decision-makers in the present century [1]. On the other hand, prediabetes can increase the risk of developing type 2 diabetes mellitus (T2DM), cardiovascular disease, periodontal disease, cognitive dysfunction, microvascular disease, hypertension (HTN), obstructive sleep apnea, metabolic syndrome, fatty liver disease, and cancer [2]. According to International Diabetes Federation, the global prevalence of T2DM and impaired glucose tolerance was reported 7.3% (4.8–11.9%) and 8.8% (7.2–11.3%) among adults (20–79 years) in 2017 and was predicted 8.3% (5.6%-13.9%) and 9.9% (7.5–12.7%) in 2045, respectively [3]. The incidence of prediabetes was reported above 4% each year, or about 32.8% among Iranian adults aged over 20 based on the findings of a 9-year cohort study in Iran [4]. In another’s 7-year and 5-year cohort studies in Isfahan (center of Iran) and Ahvaz (southwest Iran), prediabetes incidence was 32.3 [5] and 40.8 per 1000 person-years [6] among adults aged over 20, respectively. The prevalence of prediabetes and diabetes was reported 22.6 and 15.2% among Ahvaz adults over 20 years in 2009 while after 5 years (2014) reached 18.3 and 20.9% (6). In a 5-year cohort study (Ahvaz, southwest Iran), the higher prediabetes prevalence was related to male and older age [6] while in the 7-cohort study (Isfahan), gender did not play the main role in the increased risk of prediabetes [5].

The subjects with older age, living in a rural area, unhealthy diet, overweight, obesity, abnormal waist circumference (WC), high waist-to-hip ratio (WHR), low physical activity (PA), smoking habits, marital status, education levels, excessive alcohol, history of diseases (metabolic syndrome and HTN and hypertriglyceridemia), the increased levels of serum glutamic pyruvic transaminase (GPT), serum glutamic oxaloacetic transaminase (GOT), gamma-glutamyltransferase (GGT), alkaline phosphatase (ALP), and systolic blood pressure (SBP), and family history of T2DM were at an enhanced risk of prediabetes [3–5, 7, 8] and also the higher chances of developing prediabetes to T2DM [3, 5, 7]. Although there is no consensus on the risk factors of prediabetes and it remains controversial, the growing trend of prediabetes threatens global health systems [4–6, 9]. Many policies, programs, and strategies based on estimating the risk factors of prediabetes may be more effective and efficient and ultimately may lead to reducing the prevalence of prediabetes and T2DM [10].

Given that there are the modifiable various risk factors for prediabetes, that should be identified and addressed for each race and population, this cross-sectional study aimed to evaluate the prevalence of prediabetes and its risk factors- related lifestyle among adults aged 35–70 years from Hoveyzeh, Ahvaz, Iran: the baseline data from Hoveyzeh Cohort Study (HCS).

## Methods

### Study population and sampling methods

**Fig. 1:**
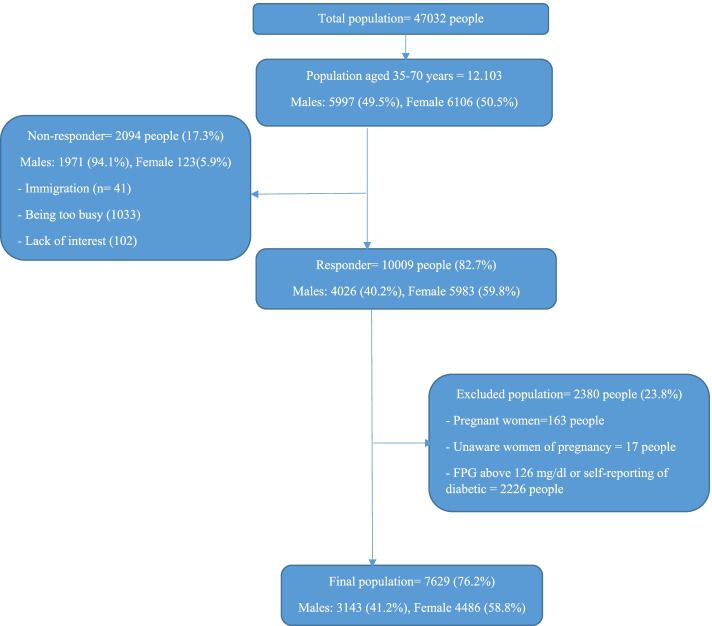
Flowchart of the participation in the present study: Hoveyzeh Cohort Study (HCS)

The present study included the baseline data from Prospective Epidemiological Research of the Iranian Adult and Neonates (PERSIAN), HCS. The subjects, who were recruited in HCS, included 10,009 out of 12,103 adults aged 35–70 years from Hoveyzeh Town (Arab community), Khuzestan Province, southwest Iran from May 23, 2016, to August 28, 2018. A total of 2094 eligibility residents (17.3% of the total population) were not enrolled due to immigration (*n* = 41), being too busy (1033), and the lack of interest (1020) in HCS. Most non-responders were male (1971). The pregnant women (*n* = 163), women who were unaware of pregnancy (*n* = 17), and diabetic subjects (*n* = 2226, based on FPG or self-reporting) were excluded from the study. The flowchart of the participation in the present study was shown in Fig. 1. Participation in HCS who signed written informed consent was voluntary. Each participant was assigned an 11-digit code (PCID) which was used to label all biological samples and documents. The details of recruitments of them were previously published [11]. Ethical approval for this study was obtained from the Ethics Committee of Ahvaz Jundishapur University of Medical Sciences (Ethical code: IR.AJUMS.REC.1399.112).

### Measurements

The demographic data (sex, age, marital status, wealth index) in past and current, PA (in the past year), sleep duration (in the past year), anthropometric data, SBP, diastolic blood pressure (DBP), and personal habits including using a cell phone (mobile), drinking alcohol habit, smoking status and drug use (past and current), history of Cardiac Ischemic (CI), Myocardial infarction (MI), and stroke were gathered through interviewer-administered questionnaires. The details of the questionnaire are available in Table S1 (Supplementary file). The PA was measured using metabolic equivalent rates (METs), which is equal to the resting metabolic rate, of self-reported daily activities of HCS participants. The daily physical activity questionnaire was used to measure MET for all participants' activities 24 h a day was calculated for all participants (Supplementary file, Table S1). A one-year food frequency questionnaire was used for estimating the mean daily energy intake. All questionnaires were completed by interviewers.

Wealth index at an individual level of socioeconomic status was calculated by the means of information on households’ possession, including freezers, TV, motorbike, cell phone, car, and vacuum cleaners, access to the internet, washing machine, computer and household utilities including house ownership, the number of rooms per capita, were entered into a principal component analysis and finally, the score of wealth index was converted to 5-ordered categories (poorest, poor, moderate, rich and richest) [12].

Anthropometric data were measured in a fasting state in the morning. Height (cm) and weight (kg) were measured using a stadiometer (Seca 206) and a standing scale (Seca 755), respectively. Waist and hip (cm) were measured using Seca locked tape meters. Body Mass Index (BMI) (kg/m^2^) was calculated as body weight (kg) divided by the square of the height (m^2^) and categorized into four groups: a) underweight: BMI < 18.5; b) normal range: BMI = 18.5 to 24.9, c) overweight: BMI = 25.0–29.9; obese: BMI > 30. The WHR was calculated by WC (cm) divided by hip circumferences (HC) (cm). Abnormal WC was defined as ≥ 102 cm in men and ≥ 88 cm in women. A healthy WHR was considered as ≤ 0.85 and ≤ 0.90 for women and men, respectively. HC was assessed at the level of the largest lateral extension of the hip.

Daily energy intake was estimated by summing daily equivalent frequency × portion size (g) × energy density (kcal/g) for each one-year food frequency questionnaire (FFQ) item (130 items in the past year).

Riester sphygmomanometers were used to measure DBP and SBP twice (10 min interval) on each arm following standard guidelines. HTN was defined as having an SBP ≥ 140 mmHg or DBP ≥ 90 mmHg, a prior clinical diagnosis of HTN, or using blood pressure-lowering medications or a self-reported diagnosis of HTN [13].

Biochemical parameters included fasting plasma glucose (FPG), liver enzymes ( GPT and GOT), and serum lipid profile (triglyceride (TG), total cholesterol (TC), and high-density lipoprotein cholesterol (HDL-C).

Blood samples were collocated by the trained laboratory staff at fasting state for about 10- 12 h. Immediately, they were centrifuged (Sigma, Germany) at 3000 rpm for 10 min to separate serum. Then, the required serum levels were measured by BT 1500 autoanalyzer (Biotecnica Instruments, Italy). Prediabetes was defined as impaired fasting glucose or FPG = 100- 126 mg/dl [14]. The normal levels of serum TG, TC, and HDL-C were considered ≤ 150 mg/dl, ≤ 200 mg/dl, ≥ 40 (in men) and ≥ 50 mg/dl (in women), respectively.

### Data analysis

The analysis of quantifiable and categorical variables was conducted by means ± standard deviations (SD) and frequency (number (%)), respectively. The Kolmogorov-Smirnoff test was used to test the normality distribution of data to determine the parametric or non-parametric test. The mean of variables was compared between prediabetic and non-prediabetic subjects by t-test. Further, the comparison between two categorical variables was presented by chi-square. The odd ratio of prediabetes was assessed by logistic regression (crude and adjusted analyses). Adjusting was considered for the potential confounders including age real, Marital status, wealth scores, smoking cigarettes, alcohol drinkers, history of CI, MI, and stroke, body mass index, PA, and daily energy intake. Both genders were separately analyzed. SPSS statistical software package, version 16.0 (SPSS, Inc, Chicago, Illinois, USA), was applied for the statistical analyses. *P* < 0.05 was considered statistically significant using two-tailed tests.

## Results

The prevalence of prediabetes was 20.9 and 24.6% in men and women, respectively. The total number of participants who met the eligibility criteria in the final analysis for the present study was 7629. The prevalence of overweight (36.7%), obesity (37.5%), prediabetes (25.7%), abnormal HDL-C (35.4%), TC ≥ 200 mg/dl (33.8%), TG ≥ 150 (39.7%), and HTN (21.3%) were common among the study participants (Supplementary file, Table S2). Male and female were 3143 (41.2%) and 4486 (58.8%), respectively. The mean ± SD age was 47.67 ± 8.95 (years). The females showed a high prevalence of obesity (45.3 vs. 26.5), abdominal obesity (85.2 vs. 31.1), and unhealthy WHR (90.7 vs. 80.6) compared to the males, significantly (*p* < 0.001) (Supplementary file, Table S3).

Table 1 showed the distribution of demographic, behavioral, and clinical characteristics among participants by gender. As it was presented in Table 1, the prevalence of prediabetes was different based on demographic, behavioral, and clinical characteristics in the two genders.

**Table 1:** The distribution of demographic, behavioral, and clinical characteristics among participants by gender

	**Male**			**Female**			**Total**		
**Variables**	**Prediabetes**	**Non-diabetic**	***P***	**Prediabetes**	**Non-diabetic**	***P***	**Prediabetes**	**Non-diabetic**	***P***
**History of CI** (Yes)	102 (13.7)	192 (8)	** < 0.001**	174 (14.3)	384 (11.7)	**0.022**	276 (14.1)	576 (10.2)	** < 0.001**
**History of MI** (Yes)	17 (2.3)	36 (1.5)	0.152	13 (1.1)	28 (0.9)	0.484	30 (1.5)	64 (1.1)	0.166
**History of Stroke** (Yes)	11 (1.5)	28 (1.2)	0.512	15 (1.2)	21 (0.6)	**0.059**	26 (1.3)	49 (0.9)	0.084
**Hypertension** (Yes)	209 (28)	380 (15.9)	** < 0.001**	384 (31.6)	653 (22)	** < 0.001**	593 (30.2)	1033 (18.2)	** < 0.001**
**Using mobile** (Yes)	706 (94.5)	2271 (94.8)	0.772	794 (65.4)	2123 (64.9)	0.751	1500 (76.5)	4394 (77.5)	0.349
**Smoking cigarette** (Yes)	288 (38.6)	979 (40.9)	0.262	105 (8.6)	218 (6.7)	**0.027**	393 (20)	1197 (21.1)	0.318
**Alcohol drinker** (Yes)	34 (4.6)	122 (5.1)	0.553	3 (0.2)	3 (0.1)	0.354	37 (1.9)	125 (2.2)	0.467
**Drug abuse** (Yes)	36 (4.8)	133 (5.6)	0.439	0	4 (0.1)	0.58	36 (1.8)	137 (2.4)	0.159
**Marital status**									
Single	8 (1.1)	28 (1.2)	0.418	64 (5.3)	195 (6)	** < 0.001**	72 (3.7)	223 (3.9)	** < 0.001**
Married	736 (98.5)	2344 (97.8)		939 (77.3)	2711 (82.9)		1675 (85.4)	5055 (89.2)	
Widow	2 (0.3)	10 (0.4)		172 (14.2)	286 (8.7)		174 (8.9)	296 (5.2)	
Divorced	1 (0.1)	14 (0.6)		39 (3.2)	80 (2.4)		40 (2)	94 (1.7)	
**Wealth Score**									
Poorest	108 (14.5)	412 (17.2)	0.149	275 (22.7)	749 (22.9)	0.065	383 (19.5)	1161 (20.5)	0.203
Poor	138 (18.5)	443 (18.5)		241 (19.9)	764 (23.3)		379 (19.3)	1207 (21.3)	
Moderate	143 (19.1)	502 (21)		258 (21.3)	602 (18.4)		401 (20.4)	1104 (19.5)	
Rich	182 (24.4)	504 (21)		234 (19.3)	610 (18.6)		416 (21.2)	1114 (19.7)	
Richest	176 (23.6)	535 (22.3)		206 (17)	547 (16.7)		382 (19.5)	1082 (19.1)	
**Triglyceride** < 150 mg/dl	349 (46.7)	1255 (52.4)	**0.007**	687 (56.6)	2276 (69.6)	** < 0.001**	1036 (52.8)	3531 (62.3)	** < 0.001**
Abnormal HDL-C*	198 (26.5)	669 (27.8)	0.511	705 (58.1)	1926 (59.5)	0.396	707 (36.1)	1992 (35.1)	0.468
**Cholesterol** < 200 mg/dl	452 (60.5)	1628 (67.9)	** < 0.001**	699 (57.6)	2207 (67.5)	** < 0.001**	1151 (58.7)	3835 (67.7)	** < 0.001**
**SBP** < 140 mmHg	677 (90.6)	2240 (93.5)	** < 0.001**	1105 (91)	3111 (95.1)	** < 0.001**	1763 (89.9)	5359 (94.5)	** < 0.001**
**DBP** < 90 mmHg	658 (88.1)	2248 (93.8)	**0.009**	1132 (93.2)	3135 (95.8)	** < 0.001**	1809 (92.2)	5375 (94.8)	** < 0.001**

*CI* Cardiac Ischemic, *DBP* Diastolic Blood Pressure *HDL-C* High-density lipoprotein-cholesterol, *MI* Myocardial infarction, *SBP* Systolic Blood Pressure

^*^For male < 40 mg/dl, for women < 50 mg/dl

Data presented by frequency (number (%)). *P*-value < 0.05 was considered significant

The prevalence of history of CI, marital status, HTN, SBP > 140 mm Hg, DBP ≥ 90 mm Hg, TG ≥ 150 mg/dl, and TC > 200 mg/dl (*p* < 0.001) was more in the participants with prediabetes compared to non-diabetes. The prevalence of history of CI, HTN, SBP > 140 mm Hg, TC > 200 mg/dl (*p* < 0.001), DBP > 90 mm Hg (*p* = 0.009), TG > 150 mg/dl (*p* = 0.007) were more common in men with prediabetes compared to the men with non-diabetes. In comparison to women with non- prediabetes, the prevalence of HTN, SBP > 140 mmHg, DBP > 90 mmHg, TG > 150 mg/dl, TC > 200 mg/dl, marital status (*p* < 0.001), history of CI (*p* = 0.022), and smoking cigarette (*p* = 0.027) was higher in women with prediabetes.

Table 2 was depicted the comparison of the mean of age, sleep duration, physical activity, energy intake, and anthropometric data between participants with prediabetes and non-diabetes by gender. As it was presented in Table 2, the mean of age, lifestyle habits, and anthropometric data were different between prediabetes and non-diabetes by gender.

**Table 2:** The comparison of mean of age, lifestyle habits, and anthropometric data between participants with prediabetes and non-diabetes by gender

	**Male**			**Female**			**Total**		
**Variables**	**Prediabetes**	**Non-diabetic**	***P***	**Prediabetes**	**Non-diabetic**	***P***	**Prediabetes**	**Non-diabetic**	***P***
**Age**	50.2 ± 9.38	47.43 ± 8.89	** < 0.001**	50.02 ± 9.08	46.4 ± 8.56	** < 0.001**	50.09 ± 9.2	46.84 ± 8.7	** < 0.001**
**Sleep duration** (hours)	7.12 ± 1.56	7.15 ± 1.6	0.632	7.94 ± 1.54	7.92 ± 1.45	0.699	7.63 ± 1.6	7.59 ± 1.56	0.407
**Daily energy** (kcal)	3577.77 ± 1092.60	3623.34 ± 1095.45	0.321	2795.54 ± 886.15	2887.08 ± 849.87	**0.002**	3093.51 ± 1041.5	3198.32 ± 1027.79	** < 0.001**
**Physical Activity** (MET)	37.52 ± 7.23	38.7 ± 7.65	**0.004**	36.39 ± 4.11	36.97 ± 4.05	**0.037**	36.82 ± 5.54	37.7 ± 5.91	** < 0.001**
**Body Mass Index** (kg/cm^2^)	28.61 ± 4.89	26.89 ± 4.5	** < 0.001**	30.85 ± 5.71	28.95 ± 5.39	** < 0.001**	29.99 ± 5.52	28.08 ± 5.13	** < 0.001**
**Waist Circumstance** (cm)	99.77 ± 12.14	95.25 ± 11.32	** < 0.001**	103.70 ± 11.81	98.99 ± 11.9	** < 0.001**	102.20 ± 12.09	97.41 ± 11.8	** < 0.001**
**Hip Circumference** (cm)	102.66 ± 9.2	100.22 ± 8.42	** < 0.001**	107.87 ± 10.48	105.62 ± 9.99	** < 0.001**	105.88 ± 10.31	103.34 ± 9.73	** < 0.001**
**Waist-to-Hip ratio**	0.97 ± 0.06	0.95 ± 0.06	** < 0.001**	0.96 ± 0.07	.94 ± 0.07	** < 0.001**	0.96 ± 0.06	0.94 ± 0.06	** < 0.001**

Data was presented by mean ± standard deviations (SD)

^†^Statistical analysis was performed using t-test

*P*-value < 0.05 was considered significant

In comparison to the participants with non-diabetes, the mean of age, BMI, WC, HC, and WHR was higher in participants with prediabetes (*p* < 0.001). Moreover, the mean PA levels and daily energy intake were lower in participants with prediabetes compared to the participants with non-diabetes (*p* < 0.001). The mean of age, BMI, WHR, HC, and WC (*p* < 0.001) were higher in men with prediabetes compared to the men with non- prediabetes. In addition, the mean PA levels were lower in men with prediabetes (38.7 ± 7.65 vs. 37.52 ± 7.23, *p* = 0.004). However, the mean of age, BMI, WHR, HC, and WC was higher (*p* < 0.001), the mean of daily energy intake was lower (*p* = 0.002) in women with prediabetes in comparison to women with non- prediabetes. Moreover, the mean PA levels were lower in women with prediabetes (36.39 ± 4.11 vs. 36.97 ± 4.05, *p* = 0.037).

The association of demographic, behavioral, biochemical, clinical, and anthropometric characteristics with the prevalence of prediabetes was depicted in Tables 3 and 4 (adjusted analysis). The results of the crude analyses were presented in the Supplementary file (Tables S4 and S5).

**Table 3:** The association of demographic, behavioral, and anthropometric characteristics with prevalence of prediabetes based on the adjusted analyses—logistic regression model among participants: The Hoveyzeh Cohort Study

Variables	Male		Female		Total	
	**▀Adjusted OR (95%CI)**	***p***	**▀Adjusted OR (95%CI)**	***P***	**▀Adjusted OR (95%CI)**	***P***
**Age**	1.04(1.03,1.05)	** < 0.001**	1.05(1.04,1.06)	** < 0.001**	1.04 (1.04,1.05)	** < 0.001**
**Marital status (Single)**						
Married	0.75 (0.33, 1.69)	0.49	0.76 (0.56, 1.03)	0.081	0.77 ( 0.58, 1.03)	0.08
Widow	0.36 (0.06, 2.04)	0.247	0.91 (0.63, 1.31)	0.598	0.91 (0.64, 1.28)	0.9
Divorced	0.22 (0.025, 2.01)	0.18	1.1 (0.67, 1.8)	0.703	1.01 (0.63, 1.61)	0.97
**Wealth Score (Moderate)**						
Poorest	0.89 (0.67, 1.18)	0.42	0.98 (0.78, 1.2)	0.84	0.97 (0.82, 1.15)	0.97
Poor	1.12 (0.85, 1.45)	0.43	0.85 (0.68, 1.06)	0.152	0.95 (0.8, 1.1)	0.95
Rich	1.04 (0.8, 1.35)	0.78	1.14 (0.91, 1.43)	0.24	1.11 (0.94, 1.3)	0.22
Richest	1.2 ( 0.94, 1.5)	0.15	1(0.8, 1.25)	0.998	1.09 (0.92, 1.29)	0.3
**Smoking cigarette (No)**						
** Yes**	1.16 (0.97, 1.38)	0.1	1.04 (0.8, 1.35)	0.76	1.11 (0.97, 1.28)	0.128
**Alcohol drinker (No)**						
** Yes**	1.06 (0.71, 1.58)	0.77	2.37 (0.45, 12.5)	0.31	1.11 (0.76, 1.62)	0.6
**History of Cardiac Ischemic (No)**						
** Yes**	1.45(1.09,1.93)	**0.01**	0.99 (0.81,1.23)	0.98	1.14 (0.96,1.34)	0.13
**History of Myocardial infarction (No)**						
** Yes**	0.89 (0.47,1.67)	0.71	0.73(0.36,1.46)	0.38	0.83 (0.52,1.33)	0.44
**History of Stroke (No)**						
** Yes**	0.91(0.44,1.88)	0.79	1.69 (0.85,3.38)	0.14	1.24 (0.76,2.04)	0.39
**Hypertension (No)**						
** Yes**	1.47 (1.19,1.81)	** < 0.001**	1.32 (1.12, 1.56)	**0.001**	1.38 (1.21, 1.57)	** < 0.001**
** Sleep duration** (h)	1.02 (0.99, 1.03)	0.352	1.01 (0.97, 1.04)	0.421	1.03 (0.99, 1.05)	0.397
**Body Mass Index (Normal)**						
Underweight	1.09 (0.55,2.16)	0.8	1.54 (0.8,3)	0.2	1.3 (0.81,2.09)	0.28
Overweight	1.7 (0.86,3.35)	0.12	2.08(1.08,3.99)	**0.03**	1.9 (1.19,3.03)	**0.007**
Obese	2.62 (1.32,5.18)	**0.006**	3.66 (1.91,7.02)	** < 0.001**	3.18 (1.99,5.07)	** < 0.001**
**Waist Circumstance (cm) (normal)**						
Abnormal*	1.026 (1.01 1.04)	** < 0.001**	1.022 (1.01, 1.03)	** < 0.001**	1.024 (1.02, 1.03)	** < 0.001**
** Hip Circumference** (cm)	1.02 (1.006, 1.04)	**0.006**	1.007 (1, 1.02)	0.63	1.01 (1.003, 1.02)	**0.008**
**Waist-to-Hip ratio (normal)**						
Abnormal**	1.059 (0.81, 1.38)	0.68	1.14 (0.88, 1.46)	0.316	1.11 (0.93, 1.33)	0.255
**Physical Activity (Quartile 1)**						
** Quartile 2**	1.18 (0.95,1.46)	0.14	0.93 (0.74, 1.165)	0.52	1.06 (0.91,1.24)	0.44
** Quartile 3**	1.24(0.97,1.59)	0.08	0.9 (0.74, 1.09)	0.28	1.05(0.9,1.22)	0.52
** Quartile 4**	1.22(0.95,1.57)	0.12	0.85 (0.7, 1.032)	0.1	0.99 (0.86,1.15)	0.94
**Daily Energy (Kcal) (Quartile 1)**						
** Quartile 2**	1.06 (0.8,1.4)	0.69	1.11 (0.89,1.39)	0.99	1.11(0.96,1.3)	0.16
** Quartile 3**	1.04 (0.83,1.32)	0.71	1(0.8,1.25)	0.76	1.01(0.87,1.18)	0.89
** Quartile 4**	0.85 (0.69,1.05)	0.13	0.96(0.76,1.22)	0.42	0.91(0.78,1.06)	0.23

^*^Abnormal waist circumstance was defined as ≥ 102 cm in men and ≥ than 88 cm in women

^**^A normal WHR was considered as ≤ 0.85 and ≤ 0.90 for women and men, respectively

*OR *Odds ratio, *CI *Confidence Interval. *P*-value < 0.05 was considered significant

▀Adjusted for age real, Marital status, wealth scores, smoking cigarette, Alcohol drinker, history of CI, MI, and stroke, Body Mass Index, PA, and daily energy intak

**Table 4:** The association of biochemical and clinical parameters with prevalence of prediabetes based on the adjusted analyses—logistic regression model among participants: The Hoveyzeh Cohort Study

Variables	Male		Female		Total	
	**▀Adjusted OR (95%CI)**	***P***	**▀Adjusted OR (95%CI)**	**p**	**▀Adjusted OR (95%CI)**	***P***
**Triglyceride (mg/dl) (≤ 150 mg/dl)**						
150-199 mg/dl	1.06 (0.85,1.33)	0.6	1.32(1.11,1.58)	**0.002**	1.32(1.16,1.51)	** < 0.001**
≥ 200 mg/dl	1.36 (1.11,1.67)	**0.03**	1.73(1.44,2.07)	** < 0.001**	1.64(1.44,1.86)	** < 0.001**
**Cholesterol (≤ 200 mg/dl)**						
200–239 mg/dl	1.18(1.01,1.44)	** < 0.001**	1.18 (1.01,1.38)	**0.042**	1.33 (1.18,1.49)	** < 0.001**
≥ 240 mg/dl	2 (1.5, 2.68)	** < 0.001**	1.53 (1.22,1.91)	** < 0.001**	2.04 (1.72,2.42)	** < 0.001**
HDL-C (Normal)*						
Abnormal*	0.91 (0.75,1.105)	0.35	1.035 (0.9,1.19)	0.63	1.04(0.93,1.16)	0.47
**Systolic Blood Pressure (≤ 140 mmHg)**						
≥ 140 mmHg	1.48 (1.11,1.98)	**0**.**008**	1.39 (1.07,1.82)	**0.014**	1.95(1.62,2.35)	** < 0.001**
**Diastolic Blood Pressure (≤ 90 mmHg)**						
≥ 90 mmHg	1.14 (0.84,1.55)	0.38	1.31 (0.98,1.76)	**0.011**	1.54(1.26,1.89)	** < 0.001**
** GOT**	1.016 (1.007,1.026)	**0.001**	1.012 (1.003,1.02)	**0.005**	1.013 (1.007,1.019)	**0.001**
** GPT**	1.009 (1.005,1.014)	** < 0.001**	1.012 (1.006,1.017)	** < 0.001**	1.01 (1.006,1.013)	** < 0.001**

*HDL-C* High-density Lipoprotein Cholesterol, *GOT* Glutamic Oxaloacetic transaminase, *GPT* Glutamic pyruvic transaminase

^*^For male: HDL-C < 40 mg/dl, for women < 50 mg/dl

Normal HDL-C is defined as HDL-C ≥ 40 (in men) and ≥ 50 mg/dl (in women)

*OR* Odds ratio, *CI* Confidence Interval

*P*-value < 0.05 was considered significant

^▀^Adjusted for all variables in Table 3 (age real, Marital status, wealth scores, smoking cigarette, Alcohol drinker, history of CI, MI, and stroke, Body Mass Index, PA, and daily energy intake)

In the adjusted analysis, there were higher odds of having prediabetes for overweight (OR = 1.9, 95% CI: (1.19, 3.03), *p* = 0.007), HC (OR = 1.01, 95% CI: (1.003, 1.02), *p* = 0.008), GOT (OR = 1.013, 95% CI: (1.007, 1.019), *p* = 0.001), older age (OR = 1.04, 95% CI: (1.04, 1.05), *p* < 0.001), obesity (OR = 3.18, 95% CI: (1.99, 5.07), *p* < 0.001), HTN (OR = 1.38, 95% CI: (1.21, 1.57), *p* < 0.001), GPT (OR = 1.01, 95% CI: (1.006, 1.013), *p* < 0.001), TG = 150 mg/dl (OR = 1.32, 95% CI: (1.16, 1.51), *p* < 0.001), TG ≥ 200 mg/dl (OR = 1.64 (95% CI: 1.44, 1.86), *p* < 0.001), TC = 200- 239 mg/dl (OR = 1.33, 95% CI: (1.18, 1.49), *p* < 0.001), TC ≥ 240 mg/dl (OR = 2.04, 95% CI: (1.72, 2.42), *p* < 0.001), DBP ≥ 90 mm Hg (OR = 1.01, 95% CI: (1.003, 1.02), p < 0.001) and SBP ≥ 140 mm Hg (OR = 1.01, 95% CI: (1.003, 1.02), *p* < 0.001).

In the adjusted analysis, there were higher odds of having prediabetes in men with obesity (OR = 2.62, 95% CI: (1.3, 5.18), *p* = 0.006), abnormal HC (OR = 1.02, 95% CI: (1.006, 1.04), *p* = 0.006), history of CI (OR = 1.45, 95% CI: (1.09, 1.93), *p* = 0.01), GOT (OR = 1.016, 95% CI: (1.007, 1.026), *p* = 0.001), GPT (OR = 1.009, 95% CI: (1.005, 1.014), *p* = 0.001), HTN (OR = 1.47, 95% CI: (1.19, 1.81), *p* < 0.001), SBP (OR = 1.48, 95% CI: (1.11, 1.98), *p* = 0.008), TG ≥ 200 mg/dl (OR = 1.36, 95% CI: (1.11, 1.67), *p* = 0.003), older age (OR = 1.05, 95% CI: (1.04, 1.06), *p* < 0.001), abnormal HC (OR = 1.04, 95% CI: (1.04, 1.05), *p* < 0.001), TC = 200- 239 mg/dl (OR = 1.18, 95% CI: 1.01, 1.44), *p* < 0.001), TC ≥ 240 mg/dl (OR = 2, 95% CI: (1.5, 2.68), *p* < 0.001) compared to non- prediabetes.

In the adjusted analysis, there were higher odds of having prediabetes in women with older age (OR = 1.05, 95% CI: (1.04, 1.06), *p* < 0.001), HTN (OR = 1.32, 95% CI: (1.12, 1.56), *p* = 0.001), overweight (OR = 2.08, 95% CI: (1.01, 1.03), *p* = 0.03), obesity (OR = 3.66, 95% CI: (1.99, 7.02, *p* < 0.001), abnormal WC (OR = 1.022, 95% CI: (1.44, 2.07), *p* < 0.001), TG = 150- 199 mg/dl (OR = 1.32, 95% CI: (1.11, 1.58), *p* = 0.002), TG ≥ 200 mg/dl (OR = 1.73, 95% CI: (1.44, 2.07), *p* < 0.001), TC = 200- 239 mg/dl (OR = 1.18, 95% CI: (1.01, 1.38), *p* = 0.042), TC ≥ 240 mg/dl (OR = 1.53, 95% CI: (1.22, 1.91), *p* < 0.001), DBP (OR = 1.31, 95% CI: (0.98, 1.76), *p* = 0.011), SBP (OR = 1.39, 95% CI: (1.07, 1.82), *p* = 0.014), and GOT (OR = 1.012, 95% CI: (1.003, 1.02), *p* = 0.005), GPT (OR = 1.012, 95% CI: (1.006, 1.017), *p* < 0.001),

## Discussion

The present study assessed the prevalence and risk factors of prediabetes in a population-based sample of Iranian adults residing in Hoveyzeh Town, Khuzestan (Arab community). The prevalence of overweight, obesity, prediabetes, dyslipidemia, and HTN was high in the Arab community which is consistent with the results of studies on populations settled in the Middle East [1, 4–6, 15, 16]. However, the prevalence of prediabetes in the present study (Hoveyzeh Town) was lower compared to the results of the previous study in Khuzestan, which showed a higher prevalence of prediabetes in Arab ethnic compared to Bakhtiari, Fars, and Turk/Kurd/Lur [16]. The prevalence of prediabetes in the present study was higher in the women compared to the men that were in disagree with the result of a study conducted by Hariri et al. [16]. It may emanate from a higher prevalence of risk factors of prediabetes (overweight, obesity, and abnormal WC and WHR) in females.

The present results showed the prevalence of prediabetes was positively related to the history of CI, HTN, marital status, and abnormal TG, TC, SBP, and DBP while there was no significant difference between the prevalence of prediabetes with history of MI and stroke, wealth score, using a mobile, smoking cigarette, alcohol drinker, and drug abuse. The prevalence of prediabetes in men was related to a history of CI, HTN, and abnormal TG, TC, SBP, and DBP. The prevalence of prediabetes in women was positively related to a history of CI, HTN, smoking cigarettes, marital status, and abnormal TG, TC, SBP, and DBP.

Moreover, the mean of age, anthropometric parameters were higher while the mean of PA levels was lower in participants as well as women and men with prediabetes. However, daily energy intake was found no significant difference in the men with prediabetes compared to non-diabetes, it was lower in total participants as well as women with prediabetes compared to non-prediabetes. Sleep duration was showed no significant difference between participants with prediabetes and no-diabetes.

An increased chance for total participants with prediabetes was related to older age, HTN, overweight, obesity, elevated biochemical parameters (TG, TC, GOT, GPT, SBP, and DBP), and abnormal WC and HC.

In men, there were greater chances for prediabetes in participants with older age, history of CI, HTN, SBP, obesity, elevated biochemical parameters (TG, TC, GOT, GPT), and abnormal WC and HC.

In women, there were higher chances of prediabetes in participants with older age, HTN, SBP, and DBP, overweight, obesity, elevated biochemical parameters (TG, TC, GOT, GPT), and abnormal WC.

Moreover, Hariri et al. [16] reported that there was a higher prevalence of prediabetes in the Khuzestan participants with HTN, abnormal WC, older age in accordance with the present findings.

The risk factors of prediabetes (age, HTN, obesity, WC, and SBP, GOT, GPT, and cholesterol higher than normal,) were relatively similar in both genders as well as the general population that was in conformity with the results of a study conducted by Hariri et al.[16]. In the present study, more risk of prediabetes did not relate to overweight, abnormal DBP, and TG (200–239 mg/dl) in the males while they were related to females and the general population.

They showed that the risk ratio of prediabetes in Khuzestan residents was related to aging, male, overweight, obesity, WC, HTN, and lower education levels [16]. The difference in biological, biochemical, physiological, environmental, behavioral, and socioeconomic status can involve gender inequalities and can impact the different prevalence of dysglycemia [17].

Some effective risk factors (genetic, epigenetic, and nutritional factors and sex hormones) which play an important role in body metabolism and function, anthropometric measurement, and inflammatory responses were not evaluated in the present and previous studies [5–7, 17]. On the other hand, psychosocial stress appears to have a more important role in women rather than men [17]. However, the previous findings showed high-energy diet is related to a higher chance of prediabetes [18, 19], our results were not shown this relationship and on the other hand, energy intake was lower among prediabetes.

It seems that energy intake alone cannot justify the prevalence of prediabetes. Many nutrition habits, including the timing of energy intake during the day, type of dietary patterns, and meal frequency associated with diabetes should also be considered. On the other hand, the subjects with prediabetes may have reduced their energy intake due to being aware of their disease [19, 20].

There was a higher chance of prediabetes in Chinese with HTN, obesity, age > 46 years, low HDL-C, high LDL-C, current smoking, and central obesity, and a higher education level [21] while the present study wasn’t shown any association between HDL-C, smoking and wealth score with prediabetes. In a study in a wealthy China rural population, SBP was the only risk factor of prediabetes while the other factors (aging, SBP, TG, and white blood cell) were associated with T2DM [7]. Age, BMI, marital status, and educational attainment were the predictors for prediabetes among males in Saudi Arabia [22]. However, the greater chances of prediabetes were related to aging, living in rural areas, unhealthy diet, overweight, obesity, waist gain, high WHR, HTN, and hypertriglyceridemia; a lower chance of prediabetes was found in participants with higher education and weight loss in three different districts of Iran [5]. In another study in Tehran, Iran [4], the development of prediabetes was related to marital status, education, and smoking habits. In the present study, some risk factors of prediabetes (older age, HTN, obesity, and LDL-C) were relatively similar to the findings of the study which conducted by Song [21]. On the other hand, our findings did not show the greater chances of prediabetes for lower HDL-C, higher education levels, and central obesity in disagreeing with the findings of Song [21].

The factors that increase the risk of prediabetes among males in Saudi Arabia included older age, obesity, overweight, being married, and less education [20] which was similar to the present results.

Despite inconsistencies in findings, most studies have shown that aging and abnormal biochemical, clinical, and anthropometric parameters including lipid profiles, liver enzymes, HTN, BMI, HC, WHR, WC) along with some socio-demographic characteristics and unhealthy lifestyle (income, education levels, gender, sleep habits, PA levels and etc.) increase the chances of prediabetes [4, 15, 18, 22–24].

### Limitations and strengths

The present study had some limitations including 1) the studied sample is restricted to the 35–70-year age group; therefore, the results cannot be generalized to younger or older age groups; 2) using only one method for diagnosis and the unavailability of oral glucose tolerance test (OGTT) and HbA1c testing might be other causes of bias of the results (underestimating the prevalence of prediabetes in this region of Iran) due to many epidemiological studies showed a significant discordance between OGTT and FBG-based prediabetes diagnoses, and the prevalence of newly diagnosed prediabetes [23]; 3) the design of the study was cross-sectional thus could not prove causal relationships between the studied risk factors and prediabetes, it may emanate from other unexplored factors; 4) the data on some habits include using mobile, smoking, drinking alcohol, and the PA was self-reported and may suffer from recall bias; 5) some risk factors related to prediabetes (timing of energy intake during the day, dietary patterns, meal frequency, and etc.) [20] were not evaluated.

Despite limitations, there were some strengths in the present study. To the best of our knowledge, the current study is the first study to specifically assess the prevalence and risk factors of prediabetes in both genders in a relatively large population of Hoveyzeh town. Further, the results of the present study may be extrapolated to a large area of southwestern Iran and southern Iraq due to having similarities in terms of race, ethnicity, culture, and lifestyle. Moreover, multiple potential risk factors enabled a comprehensive risk assessment for prediabetes. On the other hand, using multivariable logistic regression models the findings controlled for multiple potential confounders. Further, assessing daily energy intake was obtained by face-to-face interview using a validated semi-quantitative FFQ, covering 130 food and beverage items. This FFQ enabled us to obtain relatively comprehensive and reliable information daily energy intake of participants in the preceding year. Finally, our findings provided valuable information for the early prevention of prediabetes and progress to T2DM through identifying risk factors of prediabetes in an Arab population.

Given that the previous and present findings showed the risk factors of prediabetes are varied and depend on different variables such as age, ethnicity, race, gender, geographical area, and culture, comprehensive research, especially prospective studies are necessary to validate the present findings, determine risk factors in each area worldwide and evaluate the causal relationship of risk factors of prediabetes.

Unfortunately, the health decision-makers in Iran have not paid attention to the alarm of the high prevalence of prediabetes and T2DM. The results of present and similar studies may be a spark for them to make broader practical policies with constructive interventions to prevent prediabetes and T2DM.

## Conclusion

The prevalence of prediabetes, overweight, obesity, HTN, and abnormal lipid profiles was common among the Arab community (Hoveyzeh). Major determinants include aging, overweight, obesity, HTN, higher liver enzymes, and dyslipidemia, which were relatively similar in the two sexes. Our findings emphasized that prediabetes is a multifactorial disease with specific risk factors for each population, so, the determining risk factors related to each race based on gender are important in the early prevention of prediabetes.

It seems to be necessary to design the prospective studies to confirm whether a true causal association exists. Further, more researches need for evaluating sex-dimorphic pathophysiological mechanisms of diabetes and its complications. It is suggested that health policymakers set an immediate deadline for control and prevention of prediabetes based on the findings of high-quality and large sample size studies.

## Supplementary Information

**Additional file 1:** **Table S1. **General questionnaireincluding demographic, socioeconomic status, behavior habits, and medicalhistory. **Table S2. **The distribution of some variables in the study population (*n*= 7629). **Table S3.** The comparison offrequency of anthropometric data by gender. **TableS4.** The association of demographic, behavioral, andanthropometric characteristics with prevalence of prediabetes based on thecrude analyses- logistic regression model among participants: The HoveyzehCohort Study. **TableS5.** The association of biochemical and clinicalparameters with prevalence of prediabetes based on the crude analyses -logistic regression model among participants: The Hoveyzeh Cohort Study.

## Data Availability

The datasets used and/or analyzed during the current study are available from the corresponding author on reasonable request.

## Abbreviations

ALP: Alkaline phosphatase
BMI: Body Mass Index
CI: Cardiac Ischemic
DBP: Diastolic blood pressure
FFQ: Food frequency questionnaire
HCS: Hoveyzeh Cohort Study
HDL-C: High-density lipoprotein cholesterol
GGT: Gamma-glutamyltransferase
MI: Myocardial infarction
PA: Physical activity
PERSIAN: Prospective Epidemiological Research of the Iranian Adult and Neonates
SBP: Systolic blood pressure
SD: Standard deviations
GOT: Glutamic oxaloacetic transaminase
GPT: Glutamic-pyruvic transaminase
T2DM: Type 2 diabetes mellitus
TC: Total cholesterol
TG: Triglyceride
WC: Waist circumference
WHR: High waist-to-hip ratio
